# Evaluation of Providers' Assessment of Clinical History When Using the HEART Score in the Emergency Department in the Kingdom of Saudi Arabia Hospitals: A Cross-Sectional Study

**DOI:** 10.7759/cureus.93797

**Published:** 2025-10-03

**Authors:** Shrooq M Hawati, Fares Binobaid, Asmaa Alsaeigh, Walaa Alameer, Rawan M Almontashri, Adhwa Qari, Ghalia Maghrabi

**Affiliations:** 1 Emergency Medicine, Security Forces Hospital-Makkah, Makkah, SAU; 2 Emergency Medicine, King Abdulaziz Hospital-Makkah, Makkah, SAU; 3 Medicine and Surgery, Umm Al-Qura University (UQU), Makkah, SAU; 4 Emergency Medicine, King Abdulaziz Medical City, Jeddah, SAU

**Keywords:** cardiology, clinical history, cross sectional, emergency, emergency department, emergency medicine physician, heart score, saudi arabia

## Abstract

Background: Emergency physicians are frequently confronted with large amounts of information, making it challenging to objectively determine the nature of diseases. Overestimation of the HEART (history, ECG, age, risk factors, and troponin) score may lead to unnecessary testing, medication, procedures, and potentially invasive tests.

Objective: This study aims to evaluate the effectiveness of emergency medicine (EM) and cardiology providers in using the HEART score.

Method: A cross-sectional study involving EM and cardiology physicians was conducted in Saudi Arabia. A questionnaire was used to collect data about participants' demographics and work-related data, experience using the HEART score in clinical practice, opinion about the tool and its most subjective component, effect of overestimation, underestimation of the historical points of the HEART score, most common underestimated and overestimated risk factors, and evaluation of the history portion of the HEART score.

Results: Most of the participants said that they used the HEART score in clinical practice and thought that it was an effective tool for risk-stratifying patients with chest pain. The most subjective components of the HEART score were risk factors, ECG assessment, and age. The most underestimated risk factors were hypercholesterolemia, smoking, and obesity, and the most overestimated were positive family history, diabetes, and high blood pressure. When comparing significant vs. mild distress, a significantly higher percentage of EM physicians overestimated the historical portion of the HEART score compared to cardiologists. A significantly higher percentage of cardiologists underestimated the historical portion of the HEART score due to a previous negative stress test, compared to cases with no prior workup and a lower versus a higher socioeconomic status (SES). ED physicians were also significantly more likely than cardiologists to use the HEART score in clinical practice and to believe that it is a useful tool for risk-stratifying patients with chest pain. According to cardiologists, the most subjective component of the HEART score was the patient's age, whereas it was risk factors for ED physicians. Patient death following a complication was the most objective effect of underestimated historical suspicion during clinical practice for cardiologists, whereas obesity or hypercholesterolemia was the most objective effect for ED physicians. Diabetes and a positive family history were the most underestimated risk factors for cardiologists, while smoking and hypercholesterolemia were the most overestimated. Obesity and hypercholesterolemia were the most underestimated risk factors for ED physicians, while hypertension (HTN), diabetes, and a positive family history were the most overestimated.

Conclusion: Hospitals should offer educational workshops to help providers understand the benefits of incorporating the HEART score into clinical decision-making.

## Introduction

Chest pain is defined as discomfort or pain along the anterior chest wall, extending from the neck to the lower chest wall [1-4]. It is a common chief complaint, and distinguishing benign from potentially lethal causes is critical, especially in emergency departments (EDs) [1,5-10].

The HEART score is a combination of five variables: history, electrocardiogram (ECG), age, risk factors, and troponin (HEART). It is highly sensitive (with a 95% clinical prediction) and used for triaging patients with chest pain based on their risk of major adverse cardiovascular events (MACE) in the short term [11-15]. However, its sensitivity remains suboptimal. It helps to improve ED performance while lowering the costs of observation and less invasive cardiac tests [1,7,8,11,13]. The HEART score parameters are used to categorize patient's risk of MACE as low, moderate, or high. It facilitates distinction between patients with and without MACE (myocardial infarction [MI], percutaneous coronary intervention [PCI], coronary artery bypass graft [CABG], or death) [11-13,16-19].

Ischemic heart disease (IHD) is a leading cause of death in the Kingdom of Saudi Arabia, and its prevalence is increasing due to the high prevalence of cardiovascular risk factors. A key finding in acute coronary syndrome (ACS) emerged 10 years earlier in the Saudi population than in the Western population [10,14,16,20].

ED physicians frequently encounter abundant verbal and nonverbal information, including patient appearance, demographics, and cardiac risk, which can make it difficult to objectively assess chest pain [12,15,19]. This subjectivity is a key source of overestimation in patient histories and, consequently, HEART scores [9,19]. This overestimation has the potential to increase resource use and the financial burden on healthcare services and patients, ultimately compromising patient care [3,6,9,19].

Overestimation of the HEART score may predispose patients to unnecessary investigation, medication, procedures, and potentially invasive tests [5,7,9,19]. Accurate risk assessment is therefore necessary, but no study has evaluated emergency medicine (EM) and cardiology provider biases in Saudi Arabia hospitals while assigning suspicion to patient history using the HEART score [14,19]. Furthermore, it is unclear whether providers are calculating HEART scores in accordance with previous validation studies or whether they are consistently overestimating the risk due to a lack of knowledge. Overestimation could affect the calculation and accuracy of HEART scores used to predict MACE [3,5,14].

Previous studies on this topic have limitations, and there is a lack of EM data in the Kingdom of Saudi Arabia concerning how providers use the HEART score and various related variables. Large-scale studies are required to increase knowledge of this aspect of EM and to improve how cases are handled. The purpose of this study is to evaluate the use of the HEART score by providers in EM and cardiology.

## Materials and methods

This cross-sectional multi-center study was conducted from July to September 2023 in Saudi Arabia, in the EM and cardiology departments of governmental and private hospitals across multiple regions, including Makkah, Jeddah, Riyadh, Al-Madinah Al-Munawwarah, Al-Taif, Al-Baha, Al-Khobar, Dammam, and Yanbu.

The participants were selected according to the following criteria: all EM and cardiology residents, specialists, consultants, and fellows, general practitioners in the ED, and advanced practice providers in the governmental and private Saudi Arabian hospitals. The exclusion criteria were physicians on vacation, paramedics, pre-hospital emergency service providers, medical interns, and students. The sample included all EM and cardiology practitioners at Saudi Arabian hospitals who met the inclusion criteria.

The research aim requires assessment of a provider's ability to make a risk stratification based on a patient's chest pain description (i.e., the history portion of the HEART score) without being influenced by other elements (risk factors, patient age, patient sex, etc.). The standard for the risk assessment of the history portion that we used was that provided by the original HEART score study.

A score of 2 points (highly suspicious) is given for a history containing only specific features of cardiac chest pain, such as the character of the pain, onset and duration, relation with exercise, stress, or cold, localization, accompanying symptoms, and the response to sublingual nitrates. A score of 0 (slightly suspicious) is given for a history completely lacking any specific elements of cardiac chest pain. A score of 1 point (moderately suspicious) is given if the history contains a combination of specific and non-specific elements.

As the research aim is to evaluate the use of the HEART score, we considered these definitions to be objective and the gold standard. We used a supplemental materials survey developed by board-certified EM and cardiology practitioners. The survey included 24 questions, the first eight of which concerned the demographic information of the participants (age, gender, specialty, academic vs. community practice setting, number of years in practice, attending vs. fellow/resident/advanced practice provider status, current year in training, and in which state the provider practices). Questions 9-11 addressed the use of the HEART score ('Do you use it? Is it useful for risk stratification? Which component of the HEART score do you find most subjective?'). These questions helped ensure data integrity, as individuals who did not regularly use the score were considered more likely to misuse it. Questions 12-20 presented a series of clinical vignettes to illustrate the HEART score definition of slightly, moderately, and highly suspicious. Four additional questions were added to the supplementary survey, pertaining to secondary objectives (the effects related to elevated and underestimated historical suspicion, as well as most risk factors overestimated or underestimated by EM and cardiology medical providers).

Ethical approval for the study was obtained from the research ethics committee of the Ministry of Health, Makkah, Saudi Arabia, with the ethical approval number: H-02-K-076-0324-1085.

Data were analyzed using IBM SPSS Statistics for Windows, Version 26 (Released 2019; IBM Corp., Armonk, New York). To assess the association between the variables, the chi-squared test (χ²) was used for qualitative data expressed as numbers and percentages. Quantitative data were presented as means and standard deviations, and a p-value of <0.05 was considered statistically significant.

## Results

Of the 523 physicians studied, the mean age was 35.22 ± 8.8 years, and the mean years in clinical practice was 3.98 ± 7.55. Over half (55.6%) were female, and 61.6% were EM specialists. Approximately one-fifth (18.9%) were from Riyadh, and 57.6% worked in community hospitals. One-fifth (20.4%) were residents for two years (Table 1).

**Table 1:** Demographic and work-related characteristics of participating physicians (n = 523) Mean ± SD = mean and standard deviation (for continuous variables); n (%) = number and percentage (for categorical variables)

Variable	Mean ± SD or n (%)
Age (years)	35.22 ± 8.8
Number of years in clinical practice	3.98 ± 7.55
Gender	
Female	291 (55.6)
Male	232 (44.4)
Specialty	
Cardiology	201 (38.4)
Emergency medicine	322 (61.6)
Residence	
Al-Taif	52 (9.9)
Al-Baha	17 (3.3)
Al-Khobar	26 (5)
Almadinah Al-Munawwarah	56 (10.7)
Dammam	23 (4.4)
Jeddah	136 (26)
Makkah	90 (17.2)
Riyadh	99 (18.9)
Yanbu	24 (4.6)
Do you work in an academic or community hospital	
Academic (board training program)	222 (42.4)
Community hospital	301 (57.6)
Position title	
Board certified	49 (9.4)
Board eligible	35 (6.7)
Consultant	4 (0.8)
Fellow 1 year	32 (6.1)
Fellow 2 years	15 (2.9)
Fellow 3 years	4 (0.8)
Resident 1 year	96 (18.4)
Resident 2 years	154 (29.4)
Resident 3 years	76 (14.5)
Resident 4 years	42 (8)
Staff physician	16 (3.1)

Table 2 shows that the vast majority (83.9%) were using the HEART score in their clinical practice, and 79.9% mentioned that the HEART score is a useful tool to risk-stratify patients with chest pain. The components of the HEART score that were found to be most subjective were risk factors (29.3%), ECG assessment (0.9%), and age (20.7%).

**Table 2:** Distribution of the studied physicians according to their experience in using the HEART score tool in their clinical practice, their opinion about the tool, and its most subjective component (N = 523)

Variable	No. (%)
Do you use the HEART score tool in your clinical practice?	
No	84 (16.1)
Yes	439 (83.9)
Is the HEART score a useful tool to risk-stratify patients with chest pain?	
No	105 (20.1)
Yes	418 (79.9)
Which component of the HEART score tool do you find most subjective?	
Age	108 (20.7)
ECG assessment	120 (22.9)
History	87 (16.6)
Risk factors	153 (29.3)
Troponin	55 (10.5)

Table 3 shows that 29.3% of the physicians studied found that, according to historical points in the HEART score (high suspicion), overestimation leads to unwarranted management (medication). Of them, 47.5% mentioned that the most objective effect of underestimated historical suspicion during their clinical practice was patient death as a complication. The most underestimated risk factors, according to the participants, were hypercholesterolemia (67.1%), smoking (62.9%), and obesity (62.5%). The most overestimated risk factors were positive family history (65.2%), DM (63.7%), and HTN (60.8%).

**Table 3:** Distribution of the studied physicians according to their opinion about the effect of overestimation, underestimation of the historical points of the HEART score, and the most common underestimated and overestimated risk factors (N = 523) PCI = percutaneous coronary intervention; CABG = coronary artery bypass graft; CAD = coronary artery disease; MI = myocardial infarction; ECG = electrocardiogram; HTN = hypertension; DM = diabetes mellitus

Variable	No. (%)
According to historical points in the HEART score (high suspicion), overestimation leads to:	
Unwarranted investigation	92 (17.6)
Unwarranted admission	43 (8.2)
Unwarranted management (medication)	153 (29.3)
Unwarranted procedure (PCI-CABG)	148 (28.3)
Unwarranted CABG	74 (14.1)
None of the above	13 (2.5)
The most objective effects of underestimated historical suspicion during your clinical practice:	
Cardiogenic shock as a complication of CAD	132 (25.2)
Heart failure as a complication	41 (7.8)
Patient death as a complication	144 (47.5)
Worsening ischemic changes/MI on ECG as a complication	127 (24.3)
Worsening pain/discomfort as a complication	69 (13.2)
None of the above	10 (1.9)
The most underestimated risk factor	
Smoking	329 (62.9)
HTN	27 (5.2)
DM	53 (10.1)
Positive family history	56 (10.7)
Obesity	327 (62.5)
Hypercholesterolemia	351 (67.1)
The most overestimated risk factor	
Smoking	33 (6.3)
HTN	318 (60.8)
DM	333 (63.7)
Positive family history	341 (65.2)
Obesity	58 (11.1)
Hypercholesterolemia	64 (12.2)

Regarding the risk factors and variables assessed, most providers overestimated the historical portion of the HEART score in considering the history of coronary artery disease post-PCI as compared with a history of hypertension (HTN, 82.8%); 39.2% overestimated the historical portion of the HEART score related to significantly distressed appearance vs. mildly distressed; 34.8% overestimated the older vs. younger patients; and 32.7% overestimated the Caucasian vs. African American ethnicity (Table 4).

**Table 4:** Summary of performance of all providers when asked to assign the historical portion of the HEART score in the presence of the variables listed below, compared with the correct suspicion score HTN = hypertension; CAD s/p PCI = coronary artery disease status post percutaneous coronary; SES: socioeconomic status

Variable	Tendency of Respondents			p-value
	Overestimation	Underestimation	Right Estimation	
CAD s/p PCI vs. HTN	433 (82.8)	0 (0.0)	90 (17.2)	0.597
Significant vs. mild distress	205 (39.2)	97 (18.5)	221 (42.3)	0.42
Older vs. younger	182 (34.8)	122 (21.4)	229 (43.8)	0.199
Previous negative stress test vs. no prior workup	0 (0.0)	355 (69.9)	168 (32.1)	0.86
Lower vs. higher SES	0 (0.0)	359 (68.6)	164 (31.4)	<0.001
Caucasian vs. African American	171 (32.7)	89 (17)	263 (50.3)	0.487

Table 5 shows that, compared to other practitioners, a significantly higher percentage of EM physicians overestimated the historical portion of the HEART score in considering significant vs. mild distress (43.2% vs. 32.8%) (p ≤ 0.05). A significantly higher percentage of cardiologists underestimated the historical portion of the HEART score related to previous negative stress tests vs. no prior workup (74.1% vs. 64%) and lower vs. higher socioeconomic status (SES) (74.1% vs. 65.2%) (p ≤ 0.05).

**Table 5:** Comparison between emergency medicine providers and cardiologists according to their evaluation of the history portion of the HEART score χ² = chi-squared test; CAD s/p PCI, coronary artery disease status post percutaneous coronary; HTN = hypertension; SES: socioeconomic status

Variable	Specialty		χ2	p-value
	Cardiology	Emergency Medicine		
CAD s/p PCI vs. HTN			3.11	0.078
Overestimation	159 (79.1)	274 (85.1)		
Right estimation	42 (20.9)	48 (14.9)		
Significant vs. mild distress			7.66	0.022
Overestimation	66 (32.8)	139 (43.2)		
Underestimation	47 (23.4)	50 (15.5)		
Right estimation	88 (43.8)	133 (41.3)		
Older vs. younger			3.31	0.191
Overestimation	62 (30.8)	120 (37.3)		
Underestimation	50 (24.9)	62 (19.3)		
Right estimation	89 (44.3)	140 (43.5)		
Previous negative stress test vs. no prior workup			5.85	0.016
Underestimation	149 (74.1)	206 (64)		
Right estimation	52 (259)	116 (36)		
Lower vs. higher SES			4.56	0.033
Underestimation	149 (74.1)	210 (65.2)		
Right estimation	52 (25.9)	112 (34.8)		
Caucasian vs. African American			3.02	0.221
Overestimation	60 (29.9)	111 (34.5)		
Underestimation	41 (20.4)	48 (14.9)		
Right estimation	100 (49.8)	163 (50.6)		

**Figure 1:**
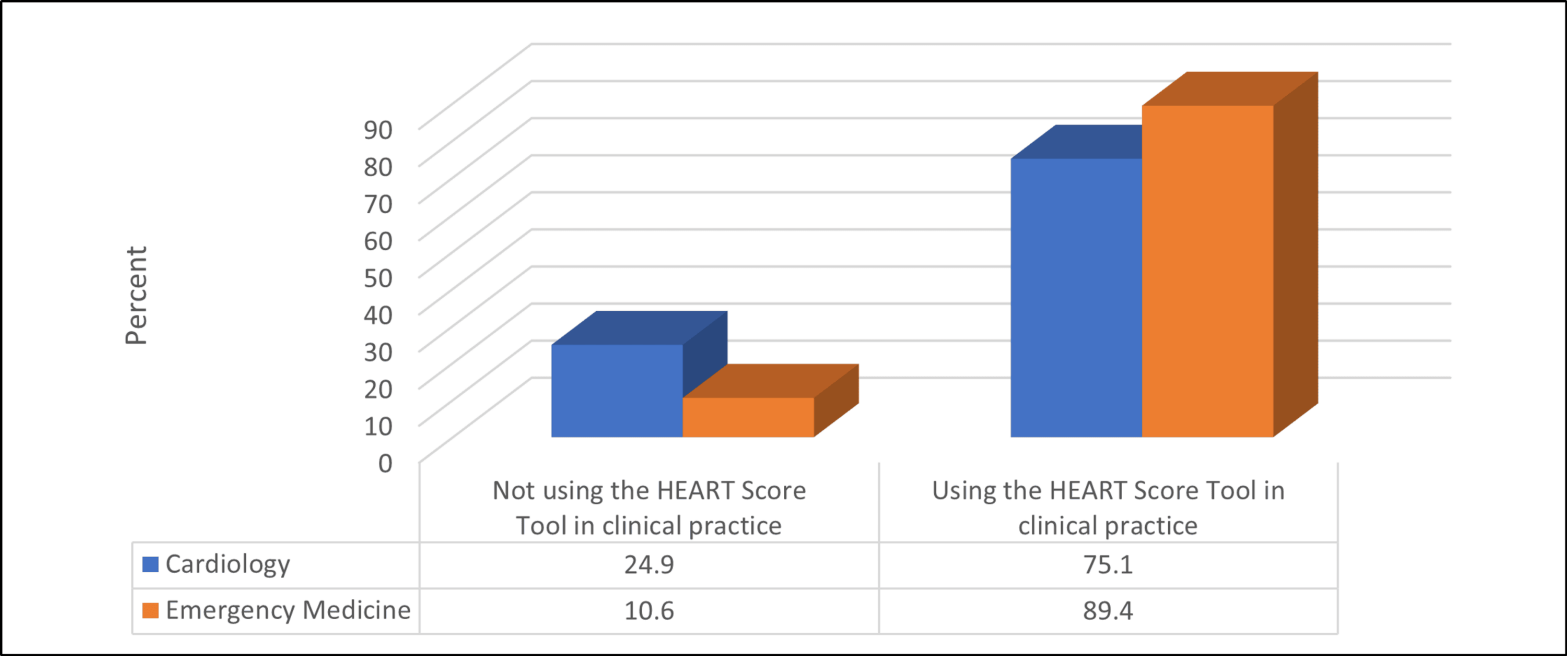
Relationship between participants' spatiality and using the HEART score tool in their clinical practice (N = 523) χ2 = 18.81, p-value = <0.001

**Figure 2:**
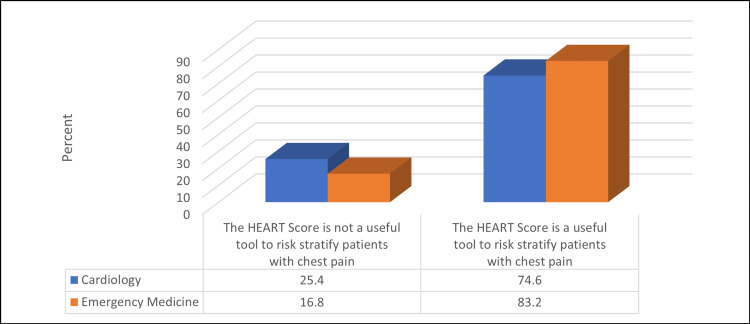
Relationship between participants' spatiality and their opinion about the usefulness of the HEART score tool to risk-stratify patients with chest pain (N = 523) χ2 = 5.7, p-value = 0.017

**Figure 3:**
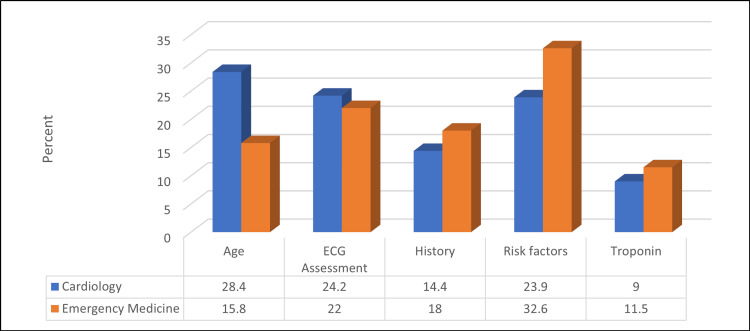
Relationship between participants' spatiality and their opinion about the component of the HEART score tool they find most subjective (N = 523) χ2 = 14.62, p-value = 0.006

Table 6 and Figures 1-3 show that a significantly higher percentage of EM physicians were using the HEART score in their clinical practice compared to cardiologists (89.4% vs. 75.1%) (p ≤ 0.05). Moreover, a significantly higher percentage of physicians thought that the HEART score was a useful tool to risk-stratify patients with chest pain compared to cardiologists (83.2% vs. 74.6%) (p ≤ 0.05). The most subjective component of the HEART score for cardiologists was patients' age (28.4%), whereas it was a risk factor for EM physicians (32.6%) (p ≤ 0.05).

**Table 6:** Comparison between emergency medicine providers and cardiologists according to their experience in using the HEART score tool in their clinical practice, their opinion about the tool, and its most subjective component (N = 523) χ² = chi-squared test

Variable	Specialty		χ2	p-value
	Cardiology	Emergency Medicine		
Do you use the HEART score tool in your clinical practice?			18.81	<0.001
No	50 (24.9)	34 (10.6)		
Yes	151 (75.1)	288 (89.4)		
Is the HEART score a useful tool to risk-stratify patients with chest pain?			5.7	0.017
No	51 (25.4)	54 (16.8)		
Yes	150 (74.6)	268 (83.2)		
Which component of the HEART score tool do you find most subjective?			14.62	0.006
Age	57 (28.4)	51 (15.8)		
ECG assessment	49 (24.2)	71 (22)		
History	29 (14.4)	58 (18)		
Risk factors	48 (23.9)	105 (32.6)		
Troponin	18 (9)	37 (11.5)		

Table 7 shows that a significantly higher percentage of cardiologists mentioned that the most objective effect of underestimated historical suspicion during their clinical practice was patient death following a complication (34.3% vs. 23.2%) (p ≤ 0.05). Furthermore, a significantly higher percentage of cardiologists reported that the most underestimated risk factors were diabetes mellitus (DM) and positive family history and reported that the most overestimated risk factors were smoking or hypercholesterolemia (p ≤ 0.05). A significantly higher percentage of EM physicians mentioned that obesity or hypercholesterolemia were the most underestimated risk factors and that HTN, DM, or positive family history were the most overestimated risk factors (p ≤ 0.05).

**Table 7:** Comparison between emergency medicine providers and cardiologists according to their opinion about the effect of overestimation, underestimation of the historical points of the HEART score, and the most common underestimated and overestimated risk factors (N = 523) PCI = percutaneous coronary intervention; CABG = coronary artery bypass graft; CAD = coronary artery disease; MI = myocardial infarction; ECG = electrocardiogram; HTN = hypertension; DM = diabetes mellitus

Variable	Specialty		χ2	p-value
	Cardiology	Emergency Medicine		
According to historical points in heart score (high suspicion), overestimation leads to:			2.44	0.785
Unwarranted investigation	31 (15.4)	61 (18.9)		
Unwarranted admission	15 (7.5)	28 (8.7)		
Unwarranted management (medication)	61 (30.3)	92 (28.6)		
Unwarranted procedure (PCI-CABG)	56 (27.9)	92 (28.6)		
Unwarranted CABG	33 (16.4)	41 (12.7)		
None of the above	5 (2.5)	8 (2.5)		
The most objective effects of underestimated historical suspicion during your clinical practice:			15.56	0.026
Cardiogenic shock as a complication of CAD	51 (25.4)	81 (25.2)		
Heart failure as a complication	16 (8)	25 (7.8)		
Patient death as a complication	69 (34.3)	75 (23.2)		
Worsening ischemic changes/MI on ECG as a complication	43 (21.4)	84 (26.2)		
Worsening pain/discomfort as a complication	17 (8.5)	52 (16.1)		
None of the above	5 (2.5)	5 (1.6)		
The most underestimated risk factor				
Smoking	92 (45.8)	237 (73.6)	41.07	<0.001
HTN	9 (4.5)	18 (5.6)	0.31	0.576
DM	28 (13.9)	25 (7.8)	5.16	0.023
Positive family history	31 (15.4)	25 (7.8)	7.59	0.006
Obesity	97 (48.3)	230 (71.4)	28.35	<0.001
Hypercholesterolemia	120 (59.7)	231 (71.7)	8.12	0.004
The most overestimated risk factor				
Smoking	22 (10.9)	11 (3.4)	11.86	0.001
HTN	97 (48.3)	221 (68.6)	21.55	<0.001
DM	102 (50.7)	231 (71.7)	23.57	<0.001
Positive family history	104 (51.7)	237 (73.6)	26.06	<0.001
Obesity	23 (11.4)	35 (10.9)	0.04	0.839
Hypercholesterolemia	33 (16.4)	31 (9.6)	5.31	0.021

## Discussion

This study aimed to determine the provider's assessment of clinical history when using the HEART score in EDs in the Kingdom of Saudi Arabia. The study involved 523 physicians with a mean age of 35.22 ± 8.8 years and a mean time in clinical practice of 3.98 ± 7.55 years. Most of the physicians were female (55.6%) and EM specialists (61.6%). This suggests that the tool is being utilized by a diverse group of healthcare professionals across various specialties, as observed in previous studies [21-23].

The HEART score is widely used by physicians, with 83.9% reporting that it is used in clinical practice. Furthermore, the HEART score is regarded as a useful tool for risk-stratifying patients with chest pain by the vast majority of physicians (79.9%). Previous studies [21,22,24,25] have also found that the HEART score is widely used by physicians, indicating a positive perception of the tool among healthcare professionals.

This study also examined the HEART score's subjective and objective components. The most subjective components identified were risk factors and ECG assessment. This suggests potential differences in how physicians interpret these factors. Furthermore, the "history" portion of the HEART score was often overestimated, particularly when patients had prior coronary artery disease (e.g., status post-PCI) or appeared significantly distressed. Previous research has found differences in risk factor perception across specialties, with cardiology physicians overestimating diabetes, positive family history, and smoking as risk factors [3,26].

In the current study, a higher percentage of overestimation was found among EM physicians for significant distress, with obesity and hypercholesterolemia being the most underestimated risk factors. These findings emphasize the significance of ongoing education and training in the use and interpretation of the HEART score across specialties [21,22,27].

Interestingly, compared to other specialties, a higher proportion of cardiologists reported observing patient death associated with underestimated historical suspicion. This indicates the potential risks associated with underestimating certain factors in patient management [21,28].

A pilot study was conducted to evaluate the implementation of the HEART score in West Balkan EDs. The study included 303 patients, with 128 classified as low-risk based on the HEART score and 175 classified as moderate-to-high-risk. Low-risk patients were younger and had fewer cardiovascular risk factors. Furthermore, for predicting MACE, the moderate-to-high-risk HEART score had a sensitivity of 91.2% (95% CI 90.2-93.4%) and a specificity of 46.5% (95% CI 39.9-48.3%) [21].

A previous study compared the agreement between HEART scores determined in clinical practice and research-generated scores, as well as their accuracy in predicting 30-day MACE. The dichotomized HEART score agreement was 78%, with the history (72%) and ECG (85%) components having the lowest agreement. Clinicians had 100% sensitivity (95% CI 88.4-100%) for MACE (vs. 86.7%, 95% CI 69.3-96.2%) and 27.8% specificity (95% CI 22.8-33.2%) vs. 34.6% (95% CI 29.3-40.3%) for researchers. According to this study, ED clinicians had only moderate agreement with research-based HEART scores [8]. These findings support the widespread use of the HEART score by emergency physicians and cardiologists as a useful tool for MACE risk stratification [3].

A cross-sectional study of EM and cardiology providers was conducted to assess bias in provider assessment of history when calculating the HEART score [19]. According to the study, most providers overestimated the historical portion of the HEART score when assessing risk factors, patient distress, age, and lower SES. Many providers underestimated history because they were aware of a previous negative stress test. When specialty was controlled for, the common theme was overestimation by EM providers and underestimation by cardiologists. Despite the presence of hypertension, gender differences, and the appearance of mild distress, cardiologists were more likely than EM providers to correctly estimate history. Cardiologists generally underestimated history due to SES considerations. All of these findings were statistically significant.

In line with the previous study [7], the current study found that EM physicians were significantly more likely to overestimate the historical portion of the HEART score when comparing significant vs. mild distress. However, cardiologists were significantly more likely to underestimate the historical portion of the HEART score compared to those with no prior workup (74.1% vs. 64%) and those with lower vs. higher SES (74.1% vs. 65.2%) [19].

The HEART score's higher sensitivity and negative predictive value make it a valuable tool for safely triaging low-risk patients to outpatient settings [21]. Nonetheless, when using the tool, healthcare professionals must be aware of any biases or misconceptions that may influence their clinical decision-making [19,21,29,30].

Overall, the study provides valuable insights into the characteristics and perceptions of physicians who utilize the HEART score. It emphasizes the tool's widespread use and positive perception among healthcare professionals while identifying areas for improvement in interpretation and understanding. These findings can help guide future research and interventions aimed at improving the use of the HEART score in clinical practice [19]. This study had the strength of a high response rate and a large sample size, which improved the generalizability of the findings.

Limitations

One limitation of the study was the short timeframe. Another limitation was the use of a cross-sectional study design, which could reveal associations between variables but not establish any causal relationships.

## Conclusions

Most participating physicians reported using the HEART score and found it useful for risk-stratifying patients with chest pain. Emergency physicians tended to overestimate, whereas cardiologists tended to underestimate the history component, with variations in perception of the most relevant risk factors. These findings highlight variability in HEART score interpretation and support the potential value of targeted educational workshops to standardize its application. Future studies should assess the impact of such interventions on clinical practice and patient outcomes.
